# Modality-specific brain representations during automatic processing of face, voice and body expressions

**DOI:** 10.3389/fnins.2023.1132088

**Published:** 2023-10-06

**Authors:** Maarten Vaessen, Kiki Van der Heijden, Beatrice de Gelder

**Affiliations:** ^1^Zuyd University of Applied Science, Maastricht, Netherlands; ^2^Radboud University, Nijmegen, Netherlands; ^3^Maastricht University, Maastricht, Netherlands

**Keywords:** multisensory affect, faces, voices, bodies, emotion perception, facial expressions, voice

## Abstract

A central question in affective science and one that is relevant for its clinical applications is how emotions provided by different stimuli are experienced and represented in the brain. Following the traditional view emotional signals are recognized with the help of emotion concepts that are typically used in descriptions of mental states and emotional experiences, irrespective of the sensory modality. This perspective motivated the search for abstract representations of emotions in the brain, shared across variations in stimulus type (face, body, voice) and sensory origin (visual, auditory). On the other hand, emotion signals like for example an aggressive gesture, trigger rapid automatic behavioral responses and this may take place before or independently of full abstract representation of the emotion. This pleads in favor specific emotion signals that may trigger rapid adaptative behavior only by mobilizing modality and stimulus specific brain representations without relying on higher order abstract emotion categories. To test this hypothesis, we presented participants with naturalistic dynamic emotion expressions of the face, the whole body, or the voice in a functional magnetic resonance (fMRI) study. To focus on automatic emotion processing and sidestep explicit concept-based emotion recognition, participants performed an unrelated target detection task presented in a different sensory modality than the stimulus. By using multivariate analyses to assess neural activity patterns in response to the different stimulus types, we reveal a stimulus category and modality specific brain organization of affective signals. Our findings are consistent with the notion that under ecological conditions emotion expressions of the face, body and voice may have different functional roles in triggering rapid adaptive behavior, even if when viewed from an abstract conceptual vantage point, they may all exemplify the same emotion. This has implications for a neuroethologically grounded emotion research program that should start from detailed behavioral observations of how face, body, and voice expressions function in naturalistic contexts.

## Introduction

In reasoning about emotion expressions and their functional and brain basis, we tend to use abstract categories and to lump together different signals presumably referring to their shared meaning. Yet the specific conditions of subjective experience of an emotional stimulus in the natural environment often determine which affective signal triggers the adaptive behavior. For example, an angry body posture alerts us already from a distance while an angry facial expression can only be seen from closer by and personal familiarity may play a role in how we react to it. Thus, the angry body expression viewed from a distance and the angry face expression seen from close by may each trigger a different reaction as adaptive behavior needs to fit the concrete context. Thus, another dimension of emotion signals besides the familiar abstract concept representation is related to their role in adaptive action. Given this essential role in triggering rapid automatic behavioral responses, sensory modality specific, local and context sensitive brain representations may play a role, suggesting that face, body, and voice expression perception may each have sensory modality specific emotion representations. Such a functional brain representation of affective signals that is sensitive to the naturalistic spatiotemporal contexts may exist besides the abstract higher order concept representations traditionally envisaged by emotion theorists (Ekman and Cordaro, 2011; but see Lindquist et al., 2012). Emotion perception in naturalistic conditions is often driven by a specific context that is relative to a behavioral goal. This specific context includes aspects that are currently not yet envisaged in human emotion research like for example the spatial parameters that matter for threat perception (de Borst et al., 2020) and convey a different behavioral status to signals from face, the body and the voice that, from an abstract vantage point, all have the same meaning. For example, since facial expressions can only be seen from sufficiently close by, decoding of facial expression may rely on processes related to personal memory more than this is the case for perception of voice or body expressions.

Previous studies comparing how the face, or the face and the voice and only in a few cases also the whole body convey emotions were motivated to find the common representations, variously referred amodal, supramodal or abstract, underlying these different expressions. They concentrated on where in the brain abstract representations of emotion categories are to be found (Peelen et al., 2010; Klasen et al., 2011). Such representations were found in high-level brain areas known for their role in categorization of mental states (Peelen et al., 2010). Specifically, medial prefrontal cortex (MPFC) and superior temporal cortex (STS) have been highlighted as representing emotions perceived in the face, voice, or body at a modality-independent level. Furthermore, these supramodal or abstract emotion representations presumably play an important role in multisensory integration by driving and sustaining convergence of the sensory inputs toward an amodal emotion representation (Gerdes et al., 2014). However, the existence of supramodal emotion representations was based on measurements of brain activity during explicit emotion recognition (Peelen et al., 2010; Klasen et al., 2011). As emotion perception in daily life is often automatic and rapid, it remains unclear whether supramodal emotion representations also emerge in the absence of explicit emotion processing.

To clarify the goal of our study it is important to distinguish the investigation of perpetual representations of emotion signals from studies on the neural basis of emotional experience, on how reward related processes are involved in processes shaping behavioral reactions and many related questions (Rolls, 2014, 2019). Clearly, perceptual representations of emotion signals do not be themselves define the emotional experience, its neural or its phenomenal basis. Other non-sensory brain areas like importantly orbitofrontal cortex are involved in defining how perception of an emotional signal ultimately gives rise to experience and behavior but these higher order issues are out of the scope of the present study.

To increase the ecological validity of our study of emotion processing in the human brain, we used naturalistic dynamic stimuli showing various emotion expressions of the body, the face or the voice. Importantly, to avoid explicit emotion recognition and verbal labeling, neither of which are normally part of daily emotion perception, participants performed a task related to another modality than that of the stimulus of interest. We used multivariate pattern analysis to identify cortical regions containing representations of emotion and to assess whether emotion representations were of supramodal nature or specific to the modality or stimulus. Following the literature we defined abstract or supramodal representation areas as regions that code what is common to a given emotion category regardless of the sensory modalities and thus have neither stimulus nor modality specific components. Our results show that during such automatic, implicit emotion processing, neuronal response patterns for varying emotions are differentiable within each stimulus type (i.e., face, body, or voice) but not across the stimulus types. This indicates that the brain represents emotions in a stimulus type and modality specific manner during implicit, ecologically valid emotion processing, as seemingly befits the requirements of rapid adaptive behavior.

## Methods

### Participants

Thirteen healthy participants (mean age = 25.3y; age range = 21–30y; two males) took part in the study. Participants reported no neurological or hearing disorders. Ethical approval was provided by the Ethical Committee of the Faculty of Psychology and Neuroscience at Maastricht University. Written consent was obtained from all participants. The experiment was carried out in accordance with the Declaration of Helsinki. Participants either received credit points or were reimbursed with a monetary reward after their participation in the scan session.

### Stimuli

**Figure 1:**
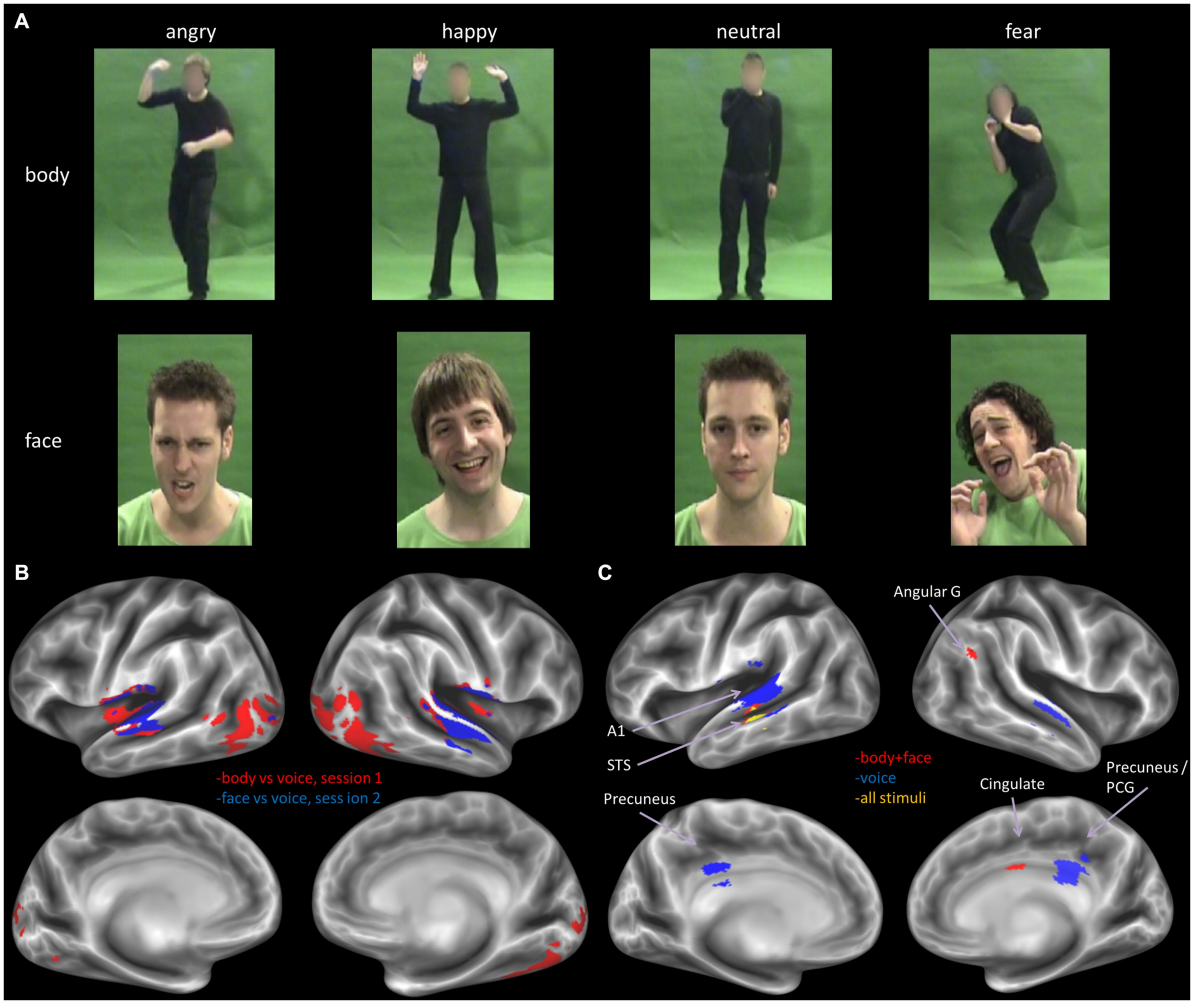
**(A)** Example stimuli from the body (top row) and face (middle row) set. The full set of stimuli can be accessed at: https://osf.io/7d83r/?view_only=a772df287d5a42d1ae7269d1eec4a14e. **(B)** Decoder trained to classify stimulus type, for the two sessions separately, results derived from thresholding the volume map at *p* < 0.05, FWE corrected. **(C)** Decoder trained to classify emotion from all stimuli (yellow), all visual stimuli (face and body, red) and all voice stimuli (blue), The displayed results are label maps derived from the volume map thresholded at *p* < 0.001 uncorrected, with a minimum cluster size threshold of *k* = 25 voxels. Angular G, angular gyrus; A1, primary auditory cortex; STS, superior temporal cortex; PCG, posterior cingulate gyrus.

Stimuli consisted of color video and audio clips of four male actors expressing three different emotional reactions to specific events (e.g., fear in a car accident or happiness at a party). Images of such events were shown to the actors during their video recordings with the goal of triggering spontaneous and natural reactions of anger, fear, happiness, and an additional neutral reaction. Importantly, the vocal recordings were acquired simultaneously with the bodily or facial expression to obtain the most natural match between visual and auditory material. A full description of the recording procedure, the validation and the video selection is given in Kret et al. (2011a), see Figure 1 top panel for several examples of the stimulus set. In total there were 16 video clips of facial expressions, 16 video clips of body expressions, and 32 audio clips of vocal expressions, half of which were recorded in combination with the facial expressions and half of which were recorded in combination with the body expressions (i.e., two audio clips per emotional expression per actor). All actors were dressed in black and filmed against a green background under controlled lighting conditions.

Video clips were computer-edited using Ulead, After Effects, and Lightworks (EditShare). For the body stimuli, faces of actors were blurred with a Gaussian mask such that only the information of the body was available. The validity of the emotional expressions in the video clips was measured with a separate emotion recognition experiment (emotion recognition accuracy >80%). For more information regarding the recording and validation of these stimuli, see Kret et al. (2011a,b).

### Experimental design and behavioral task

In a slow-event related design, participants viewed series of 1 s video clips on a projector screen or listened to series of 1 s audio clips through MR-compatible ear buds (Sensimetrics S14) equipped with sound attenuating circumaural muffs (attenuation >29 dB). The experiment consisted of 12 runs divided over 2 scan sessions. Six runs consisted of face and voice stimuli, followed by six runs consisting of body and voice stimuli. Each run was split in two halves where either *auditory* (consisting of 18 audio clips) or *visual* (consisting of 18 video clips) stimuli were presented. The 18 trials within each run half comprised 16 regular trials (consisting of 4 times 3 different emotion expressions and 4 times one neutral expression,) with an inter-stimulus interval of 10.7–11.3 s and two catch trials requiring a response. This design of using an orthogonal task was selected to divert attention from the modality of interest by blocking explicit recognition of the emotional expression and tap into automatic perception of the affective content (Vroomen et al., 2001; Rolls et al., 2006). The task instructions stipulated whether attention was to be allocated to the visual or to the auditory modality. During visual stimulus presentations, participants were instructed to detect an auditory change, and during auditory stimulus presentations, participants were instructed to detect visual change catch trials. For the auditory catch trial task, a frequency modulated tone was presented and participants had to respond whether the direction of frequency modulation was up or down. For the visual distractor task, participants indicated whether the fixation cross turned lighter or darker during the trial. A separate localizer session was also performed where participants passively viewed stimuli of faces, bodies, houses, tools and words in blocks; see Zhan et al. (2018) for details.

### Data acquisition

We measured blood-oxygen level-dependent (BOLD) signals with a 3 Tesla Siemens Trio whole body MRI scanner at the Scannexus MRI scanning facilities at Maastricht University (Scannexus, Maastricht). Functional images of the whole brain were obtained using T2*-weighted 2D echo-planar imaging (EPI) sequences [number of slices per volume = 50, 2 mm in-plane isotropic resolution, repetition time (TR) = 3,000 ms, echo time (TE) = 30 ms, flip angle (FA) = 90°, field of view (FoV) = 800 × 800 mm^2^, matrix size = 100 × 100, multi-band acceleration factor = 2, number of volumes per run = 160, total scan time per run = 8 min]. A three-dimensional (3D) T1-weighted (MPRAGE) imaging sequence was used to acquire high-resolution structural images for each of the participants [1-mm isotropic resolution, TR = 2,250 ms, TE = 2.21 ms, FA = 9°, matrix size = 256 × 256, total scan time = 7 min approx.]. The functional localizer scan also used a T2*-weighted 2D EPI sequence [number of slices per volume = 64, 2 mm in-plane isotropic resolution, TR = 2,000 ms, TE = 30 ms, FA = 77, FoV = 800 × 800 mm^2^, matrix size = 100 × 100, multi-band acceleration factor = 2, number of volumes per run = 432, total scan time per run = 14 min approx.].

### Analysis

#### Pre-processing

Data were preprocessed and analyzed with BrainVoyager QX (Brain Innovation, Maastricht, Netherlands) and custom Matlab code (Mathworks, United States) (Hausfeld et al., 2012, 2014). Preprocessing of functional data consisted of 3D motion correction (trilinear/sync interpolation using the first volume of the first run as reference), temporal high pass filtering (thresholded at five cycles per run), and slice time correction. We co-registered functional images to the anatomical T1-weighted image obtained during the first scan session and transformed anatomical and functional data to the default Talairach template.

#### Univariate analysis

We estimated a random-effects General Linear Model (RFX GLM) in BrainVoyager 21.2^1^ with a predictor for each stimulus condition of interest (12 conditions in total): four emotion conditions times three stimulus categories (face, body, voice). Additionally, we included predictors for the trials indicating the start of a new block and the catch trials. Predictors were created by convolving stimulus predictors with the canonical hemodynamic function. Finally, we included six motion parameters resulting from the motion correction as predictors of no interest. For this analysis, data was spatially smoothed with a 6 mm full-width half-maximum (FWMH) Gaussian kernel. To assess where in the brain the two different experimental factors had an influence, an ANOVA was run with either modality or emotion as a factor.

#### Multivariate analysis

We first estimated beta parameters for each stimulus trial and participant with custom MATLAB code by fitting an HRF function with a GLM to each trial in the time series. These beta values were then used as input for a searchlight multivariate pattern analysis (MVPA) with a Gaussian Naïve Bayes classifier (Ontivero-Ortega et al., 2017) which was also performed at the individual level. The searchlight was a sphere with a radius of five voxels. The Gaussian Naïve Bayes classifier is an inherently multi-class probabilistic classifier that performs similar to the much-used Support Vector Machine classifier in most scenarios, but it is computationally more efficient. The classifier was trained to decode (1) stimulus modality (visual or auditory); (2) stimulus emotion (e.g., fear in all stimulus types vs. angry in all stimulus types); (3) within-modality emotion (e.g., body angry vs. body fear); (4) cross-modal emotion (e.g., classify emotion by training on body stimuli and testing on the voice stimuli from the body session). As the GNB classifier is multi-class by design, the accuracies are calculated as the ratio of correct predictions to the total number of predictions regardless of class.

Classification accuracy was computed by averaging the decoding accuracy of all folds of a leave one-run out cross-validation procedure. We tested the significance of the observed decoding accuracies at the group level with a one-sample *t*-test against chance-level and corrected accuracy maps from each participant for multiple comparisons with the SPM Family Wise Error (FWE) procedure at *p* < 0.05 (Penny et al., 2011) (modality chance level = 50%, corresponding to two classes; emotion chance level = 25%, corresponding to four classes) with SPM12.^2^ For visualization purposes, the group volume maps were mapped to the cortical surface. As this operation involves resampling the data (during which the original statistical values get lost), surface maps are displayed with discrete label values instead of continuous statistical values. Therefore, we do not include a color bar in the surface map figures.

An additional permutation analysis was performed to further investigate the robustness of the decoding results. In this method, decoding accuracies can be compared to results from permuted emotion labels (i.e., random decoding accuracies) at the *group level*. To accomplish this, we generated a set of randomized results (*n* = 100), by randomly permuting the emotion labels (these randomizations were the same for all participants). For each of the randomizations, the decoding accuracies were calculated for each participant by repeating the full leave one-run out cross-validation procedure of the main method at the single subject level with the randomized labels. These accuracy maps were then tested against the true label accuracies by performing a paired sample *t*-test over participants. By using a paired *t*-test at the group level, we are able to assess how the true distribution of the decoding accuracies compare to random (supposedly chance level) accuracies at each voxel. Note that this method differs from the standard permutation method where the number of occurrences of the true accuracy being higher than the randomly sampled ones is counted (resulting in a *p*-value for each participant and voxel), but that we use it here because it enables assessing true vs. random accuracies directly at the group level. At the final stage, the t-maps were averaged for all participants resulting in a single averaged *t*-value group map, and thresholded at *t* > 3.9. This map was then qualitatively compared to the t-map of the main analysis described above.

#### ROI analysis

Lastly, to gain more insight into details of the responses regions known to be relevant for (emotional) sensory processing (V1, fusiform gyrus, EBA, A1 and amygdala), as well as regions known to be important for emotion or multi-modal integration: the STS and mPFC (Peelen et al., 2010), we extracted beta values from these ROIs.

Specifically, we used data of an independent localizer to identify, early visual cortex, rEBA, and rFFA. Multi-sensory regions pSTS and mPFC were located based on an anatomical definition of these areas. That is, we utilized a spherical ROI with a radius of 5 voxels centered on the reported cluster peak locations in Peelen et al. (2010). Finally, the amygdala was identified using an anatomic definition from the SPM Anatomy toolbox atlas where left and right Amygdala were combined in one ROI (Eickhoff et al., 2007). Early auditory cortex was defined bilaterally anatomically by a 5 mm sphere at the peak location for A1 reported in Warrier et al. (2009).

See Table 1 for details on location and size of the ROIs. From the ROI’s we made several plots that (1) display the mean beta values for each of the 12 conditions; (2) display the multivoxel representational dissimilarity matrix (Kriegeskorte et al., 2008) constructed by calculating the 1-r (=Pearson’s correlation coefficient) distance metric for all pairs of category averaged stimuli. The decoding accuracy of all the ROI voxels for stimulus modality, taken as the average of the accuracies for decoding of body vs. voice (of the body session) and face vs. voice (of face session) conditions is presented in Table 2. This table also includes the decoding accuracies for emotion from all stimuli, voice, face and body separately, and the crossmodal decoding accuracies for training the classifier on one modality (e.g., body emotion) and testing on the voice modality. Statistical results in this table are FDR corrected (Benjamini and Hochberg, 1995).

**Table 1:** Size and location of the ROIs.

ROI	Size	*x*	*y*	*Z*
FFA	1,784	39	−52	−26
EV	4,248	−12	−94	−8
EBA	5,488	45	−67	7
A1 L/R	6,152	−57/56	−17/−17	5/8
MPFC	2,976	11	48	17
pSTS	3,256	−47	−62	8
Amygdala L/R	784	−21/20	−6/−6	−10/−9

Size is in mm^3^, location is in MNI coordinates.

**Table 2:** Decoding accuracies group level *p*-values (FDR corrected) against chance level for the tested ROIs.

	Modality	Emotion	Emotion Body	Emotion Face	Emotion voice session 1	Emotion voice session 2	Emotion body ≥ voice	Emotion voice ≥ body	Emotion face ≥ voice	Emotion voice ≥ face
V1	0.0035	0.5235	0.5854	0.7936	0.5235	0.7002	0.7936	0.8732	0.0968	0.7002
FFA	0.0035	0.7936	0.713	0.893	0.1088	0.6185	0.6203	0.8732	0.7002	0.8147
EBA	0.0018	0.0661	0.3916	0.0968	0.7002	0.7002	0.8818	0.8818	0.7936	0.7936
A1	0	0.0445	0.9008	0.7936	0.2069	0.0168	0.1773	0.8732	0.7002	0.6203
MPFC	0.4624	0.7002	0.5477	0.713	0.5124	0.8392	0.7002	0.7936	0.5235	0.7002
pSTS	0.0445	0.8247	0.7466	0.7936	0.7009	0.7466	0.7002	0.7936	0.5124	0.7002
LR amy	0.689	0.6203	0.7936	0.8871	0.5854	0.0661	0.7002	0.168	0.5656	0.0968

Beta values of responses to the stimuli were extracted from an ROI and used to train and test a decoder on stimulus type and emotion from all stimuli or from a specific type and for crossmodal decoding. For these the header reading x ≥ y (e.g., body ≥ voice) indicated that the decoder was trained on body emotion and was tested on voice stimuli. The table shows uncorrected *p*-values as well an indication (*) where the *p*-value is significant at *p* < 0.05 after FDR correction.

## Results

### Modality-specific processing of face, body, and voice stimuli

First, we performed a univariate analysis of sensory-specific (i.e., visual vs. auditory) and emotion-specific neural processing. Using beta values estimated with an RFX GLM on the entire data set (see Methods), we ran an ANOVA with factors “stimulus type” and “emotion.” Our results did not show an interaction between emotion and stimulus type (*p* > 0.01, FDR corrected for multiple comparisons). However, as expected, the F-map for stimulus type (Supplementary Figure S1) revealed significant activation clusters (*p* < 0.01, FDR corrected for multiple comparisons) with differential mean activation across stimulus types in primary and higher-order auditory and visual regions, as well as in motor, pre-motor and dorsal/superior parietal cortex. We did not observe significant activation clusters for emotion (*p* > 0.01, FDR corrected). We then tested whether emotions were processed differentially within stimulus type, i.e., for faces, bodies and voices separately. These ANOVAs did not reveal an effect of emotion for any stimulus type (*p* < 0.01, FDR corrected).

### Multivariate pattern analysis of modality related emotion processing

Next, we performed a more sensitive multivariate pattern analysis (MVPA) using a searchlight approach (Ontivero-Ortega et al., 2017; see Methods). We sought to confirm the results of the univariate analysis and to assess whether more fine-grained emotion representations could be revealed either within a specific stimulus type or in a supramodal manner. To this end, we began by training the MVP classifier to decode stimulus type, i.e., visual vs. auditory stimuli. The classifier was trained separately for the two sessions: body vs. voice (session 1) and face vs. voice (session 2). In line with expectations, the classifier could accurately identify stimulus type in auditory and visual sensory cortices (chance level is 50%, *p* < 0.05, FWE corrected for multiple comparisons). In addition, we found clusters of above chance level decoding accuracy in fusiform cortex and large parts of the lateral occipital and temporo-occipital cortex, presumably including the extrastriatal body area (EBA; see Figure 1 bottom panel).

We then tested whether the MVP classifier trained to discriminate emotions on all stimulus types (i.e., face, body and voice combined) could accurately identify emotion from neural response patterns. Yet, like the results of the univariate analysis, we did not find classification accuracies above chance at the group level (that is, at *p* < 0.05, FWE corrected). Thus, while neural response patterns for stimulus type could be differentiated well by a MVP classifier, we did not observe a similarly robust, differentiable representation of emotions across stimulus types.

We then proceeded to evaluate our main hypothesis using the MVP classifier, that is, whether emotion specific representations emerge within modality specific neural response patterns. Specifically, we trained and tested the classifier to decode emotions within a specific stimulus modality. The visual stimulus modality consisted of the combined face and body stimuli and the auditory modality of the voice stimuli. Within the visual modality (i.e., faces and bodies), the classifier could accurately (although not significantly, based on *p* < 0.05 FWE corrected) discriminate emotions in STS, cingulate gyrus and angular gyrus (*p* < 0.001 uncorrected, cluster size threshold = 25), but not in visual cortex. Within the auditory modality (i.e., voices), the classifier discriminated emotions accurately in primary and secondary auditory cortical regions (including the superior temporal gyrus [STS], and in the precuneus (Figure 1, bottom panel and Table 3) at *p* < 0.001 uncorrected, cluster size threshold = 25). When the classifier was trained and tested to decode emotion within a specific visual stimulus type, that is, either the face stimuli or body stimuli, classification performance was at chance level.

**Table 3:** Results for the decoding of emotion.

	Cluster size	Cluster p(unc)	Peak T	Peak p(unc)	*x*	*y*	*z*
**All stimuli**							
STS L	38	0.0536	5.8422	0.0000	−62	−34	2
**Voice stimuli only**							
Planum temporale L	749	0.001	6.462	0.000	−58	−34	6
Posterior cingulate gyrus/precuneus	187	0.058	5.654	0.000	8	−34	30
Planum temporale R	210	0.046	5.206	0.000	62	−18	2
**Face and body stimuli**							
STS L	59	0.0200	6.8069	0.0000	−54	−26	−4
Cingulate gyrus R	92	0.0052	6.5513	0.0000	16	−12	20
Angular gyrus R	27	0.0979	6.4339	0.0000	60	−50	34

Additionally, we performed a permutation analysis on the obtained classification results to assess the robustness of the decoding results (see Methods). The permutation test confirmed that the classifier can accurately discriminate emotions (compared to randomized stimulus labels) based on neural activity patterns in response to stimuli in the auditory modality (voices). For the visual stimulus modality, the permutation analysis yielded slightly different results. More specifically, the classifier could discriminate emotions accurately (compared to randomized stimulus labels) only within either face stimuli (in STS and PCC) or body stimuli (in ACC), but not when trained or tested on all visual stimuli combined (Supplementary Figure S4). Classification results for the classifier trained and tested on all sensory modalities (i.e., voice, face and body) were not above the accuracy levels of the randomized stimulus labels in any region. The results show that in this study no evidence was found for visual–auditory modality independent representations.

Finally, we performed an additional MVP classification analysis to evaluate whether supramodal emotion processing regions can be identified in the brain by training a classifier to decode emotion across stimulus modalities. Being able to predict emotion by training on one modality and testing on another modality would be a strong indication of supramodal emotion encoding in the brain. Therefore, the cross-modal classifier was trained (or tested) on either the body or face stimuli and tested (or trained) on the voice stimuli from the body or face session, respectively. Thus, four whole-brain searchlight classifiers were trained in total (training on body and testing on voice, training on voice, and testing body, training on face and testing on voice, training on voice and testing on face). In contrast to the successful and significant decoding of modality and the successful decoding of emotion within stimulus type, none of these cross-modal classifiers resulted in accurate decoding of emotion (*p* < 0.05 FWE corrected, as well as at *p* < 0.001 uncorrected with cluster size threshold = 25).

### Assessing emotion representations in neural response patterns in regions of interest

**Figure 2:**
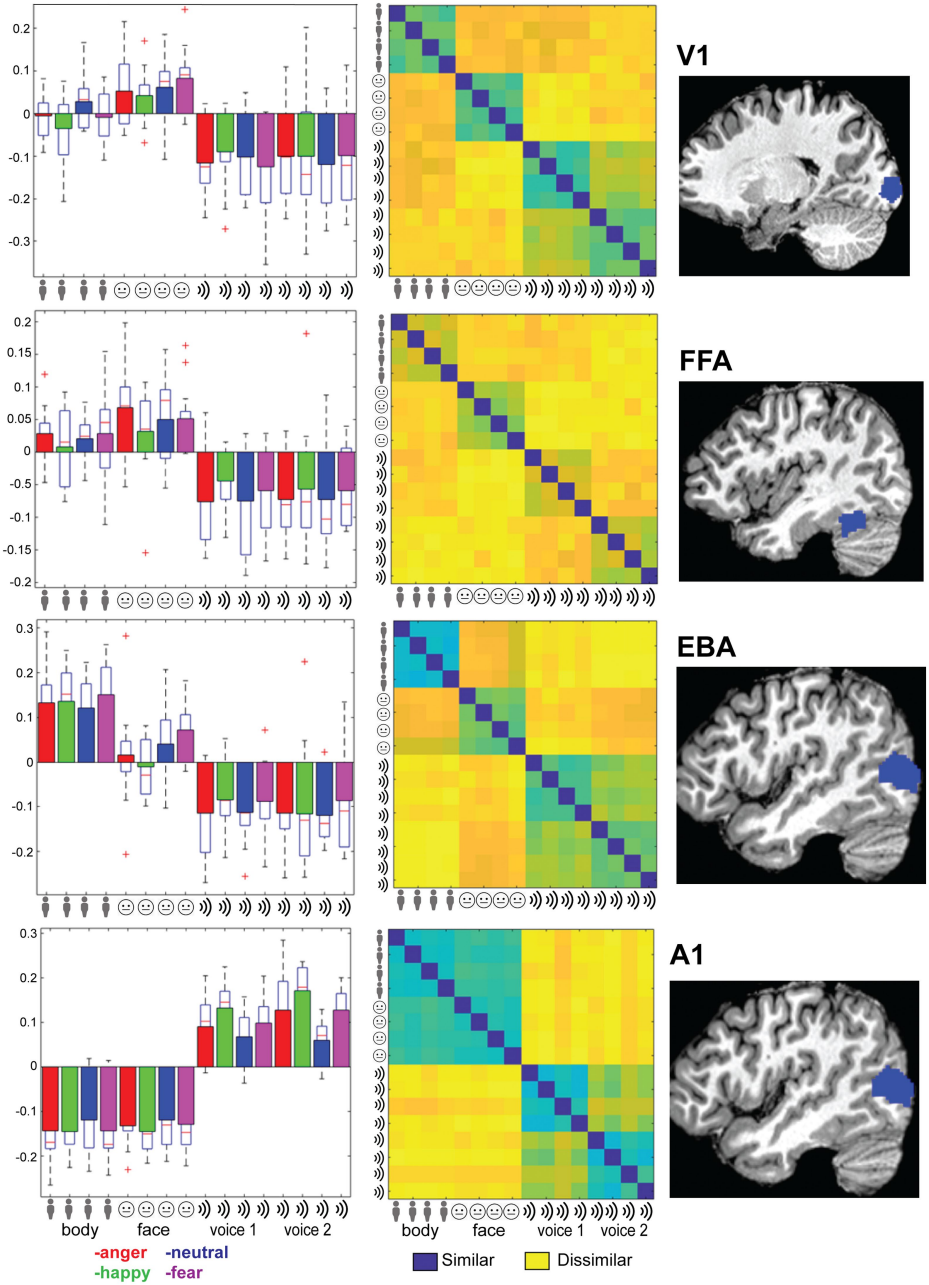
Right column: Location of the ROI (see main text for abbreviations). Left column: Trial-wise beta values for all 16 conditions: 4xtype (body, face, voice session 1 and voice session 2) and 4x emotion (indicated by color: anger-happy-neutral-fear) averaged over the ROI and group. Error bars indicate SE. Middle column: Representational dissimilarity matrix for the ROI. Blue colors indicate that responses to a pair of categories were similar (1-Pearson’s r), yellow colors indicate dissimilarity. The matrices are ordered from top-left to bottom-right in blocks of 4 by 4 for the emotional expressions in order of angerhappy-neutral-fear. Blocks are ordered by stimulus type as indicated by the icons and text: body, face, voice session1 and voice session 2.

As a last step, we evaluated whether primary sensory regions (A1 and V1) and regions that have been previously implicated in emotion processing (fusiform gyrus, EBA, amygdala) or in multi-modal regions implicated in supramodal emotion processing (pSTS and mPFC; Peelen et al., 2010) contained emotion processing using a more sensitive region-of-interest (ROI) analysis. In line with our approach in the previous analyses, we began by testing whether the classifier can discriminate sensory modality (visual vs. auditory) in the ROIs. The classifier could accurately identify the sensory modality of a stimulus in sensory cortices—as expected—but also in pSTS (*p* < 0.05, FDR corrected). We then assessed whether the classifier was able to discriminate emotion when trained and tested on all modalities together (face, body, voice). The classifier was successful only in primary auditory cortex (A1). Like our previous results, when the classifier operated on the data of each sensory modality in isolation, decoding accuracies were above chance level for voice session 2 emotion in A1. Emotion could not be decoded above chance level in the supramodal regions (mPFC and pSTS). Thus, while visual and auditory stimuli elicit distinct neural response patterns in the ROIs that can robustly be discriminated by a MVP classifier, the emotions used here do not elicit specific neural response patterns. A representational dissimilarity matrix (RDM) analysis confirms that most ROIs considered here exhibit a strong modulation by sensory modality, except for mPFC and amygdala. However, no robust emotion representations were observed in these ROIs (Figure 2, Supplementary Figure S2).

## Discussion

This study addressed a central question in the emotion literature that arises once investigations of human emotion expressions move beyond the traditional focus on facial expression. Our goal was to investigate whether implicit, ecologically valid processing of emotion expressions in either the face, the voice or the whole body is specific for the stimulus category. We used MVPA to find evidence of abstract representations that would be common to the different stimulus modality categories. Our results show that the brain contains several regions with emotion-specific representations. Importantly, in the absence of explicit emotion recognition instructions these emotion-specific representations were restricted to the respective stimulus modality or category (i.e., face, body, voice) and we did not find evidence for abstract emotion representation. Our design was entirely motivated by the focus on the sensory processes useful for emotion decoding and not on the more traditional question of how emotions are represented in the brain or how emotions are subjectively experienced. In line with this, we opted for a task that turned attention away from the stimulus presented. One may argue that the fact that we did not find modality general results for the emotion categories *per se* might have been due to this distracting task. But this was exactly the purpose as we wanted to focus on attention independent processes.

Our study presents a novel approach to the neural mechanisms underlying implicit emotion processing because we used naturalistic stimuli from three different categories and a specific task paradigm that focused on implicit emotion processing. Our results, converging across analysis techniques, highlight the specific contributions and the neural basis of emotional signals provided by each sensory modality and stimulus category. In a departure from the few previous studies using a partially comparable approach, we found evidence for sensory specific rather than abstract supramodal representations during implicit emotion processing (different from recognition or experience) that sustain perception of various affective signals as a function of the modality (visual or auditory) and the stimulus category (face, voice, or body).

To understand our findings against the background of the literature, some specific aspects of our study must be highlighted. We used dynamic realistic face and body stimuli instead of point light displays or static images. The latter are also known to complicate comparisons with dynamic auditory stimuli (Campanella and Belin, 2007). Next, our stimuli do not present prototypical emotion representations obtained by asking actors to portray emotions but present spontaneous face, voice and whole body reactions to images of familiar events. The expressions we used may therefore be more spontaneous and trigger more sensorimotor processes in the viewer than posed expressions. Third, many previous studies used explicit emotion recognition (Lee and Siegle, 2012), passive viewing (Winston et al., 2003), implicit tasks like gender categorization (Dricu and Fruhholz, 2016) or oddball tasks presented in the same modality as the stimulus. In contrast, our modality specific oddball task is presented in the alternate modality of the stimulus presentation thereby diverting attention not only from the emotion content but also from the perceptual modality in which the target stimuli of that block are shown. We discuss separately the findings on the major research questions. However, before turning to detailed results we clarify whether a different definition of amodal representation than adopted here and in the literature influences the conclusions.

### Amodal or supramodal representations

A supramodal representation of emotions in the brain is presumably a brain regions that exhibits activity patterns specific to the abstract meaning of the stimulus but that are independent from the sensory modality (visual, auditory) and the stimulus category (face, body, voice) as in Peelen et al. (2010). These authors speculated these areas possibly host abstract emotion coding neurons (Peelen et al., 2010). More recently, Schirmer and Adolphs (2017) also relate processing of face and voice expressions in pSTS and PFC to abstract emotion representations and emotional meaning.

However, it is important to underscore that those previous findings about amodal representations were obtained with an explicit recognition task that is very different from the orthogonal task we used here. Results obtained in emotion experiments are closely linked to the task used and this is certainly the case for measures of amygdalae activity (De Gelder et al., 2012). For example, in a recent study addressing the issue of explicit vs. implicit emotion tasks we found major differences in the brain representation of body emotion expressions as a function of explicit vs. implicit recognition (Marrazzo et al., 2020). Furthermore, in older studies of our group that do not use MVPA we already systematically found that the similarities and differences between brain representation of face and body expressions is a function of the specific expression just as well as the stimulus category (Van de Riet et al., 2009; Kret et al., 2011a).

To put this issue in a boarder context, the notion of supramodal emotion representation has close conceptual links with the traditional theories of human emotions and mental states. Future experiments may start from other emotion theories focusing less on mental states and more on behavior and emotions as adaptive actions (de Gelder and Poyo Solanas, 2021). For example, it has long been argued that an action theory as opposed to a mental state theory of emotions (Frijda, 1987; De Gelder et al., 2004) implies a different picture of how the brain sustains emotional processes that the traditional notion of the six predefined basic emotions going back to Ekmans’ emotion theory.

In the domain of emotion studies support for modality independent or abstract representations goes back to Ekmans’ views but another area where this issue is debated is in studies on sensory deprivation. Studies of face, voice and whole body expressions in populations with sensory deprivation are very important to clarify and substantiate our findings underscoring the importance of category and modality specificity. Currently very few studies are available that address this issue. Interestingly, there is evidence for residual perception of face, body and voice perception in patients with cortical blindness following partial or complete striate cortex lesion (reviewed in Tamietto and De Gelder, 2010). These findings underscore the role of subcortical structures and draw attention to the fact that the issue is broader than that of cortical plasticity. Important as it is, this discussion is outside the scope of the present report.

An issue that is directly relevant is the following. Based on the literature it is not yet clear what findings for example from congenital blindness would constitute clear and direct evidence for abstract representations. The finding that congenitally blind individuals react to happy speech sounds by smiling (Arias et al., 2021) provides evidence that production of facial expressions does not require learning. However, by itself this does not constitute evidence for abstract representations. Nor does it show that such abstract representations need to play a crucial role in triggering facial expressions when a congenitally blind individual is exposed to smiles in speech. Indeed, production of smiles in reaction to “hearing” smiles can be explained parsimoniously by auditory-motor associations. Such an explanation does not require any crucial appeal to high order abstract representations. Undoubtedly, the many ways in which the brain processes information input from the different sensory systems is likely to involve also abstract layers of representations, depending or not on language. But their explanatory value is dependent on task settings and stimulus context.

### Univariate analysis

Although our goal was to characterize neural responses with MVPA techniques, for the sake of comparisons with the literature, we also briefly discuss our univariate results. How do these results compare to findings and meta-analyses in the literature? In fact, there are no previous studies that used comparable materials (four emotion categories, three stimulus types, two modalities) and a different modality centered task as done here. The studies that did include bodies used only neutral actions, not whole body emotion expressions (Dricu and Fruhholz, 2016) except for one study comparing face and body expression videos by Kret et al. (2011a). Only the study by Peelen et al. used faces, bodies, and voices, but with a very different task as we discuss below (Peelen et al., 2010).

Compared to the literature, the findings of the univariate analysis present correspondences as well as differences. A previous study (Kret et al., 2011a) with face and body videos used only neutral, fear and anger expression and a visual oddball task. They reported that EBA and STS show increased activity to threatening body expressions and FG responds equally to emotional faces and bodies. For the latter, higher activity was found in cuneus, fusiform gyrus, EBA, tempo-parietal junction, superior parietal lobe, as well in as the thalamus while the amygdala was more active for facial than for bodily expressions, but independently of the facial emotion. Here we replicate that result for faces and bodies and found highly significant clusters with differential mean activation across stimulus types in primary and higher-order auditory and visual regions, as well as in motor, pre-motor and dorsal/superior parietal cortex (Supplementary Figure S1). Regions sensitive to stimulus category were not only found in primary visual and auditory cortex as expected but also in motor, pre-motor and dorsal/superior parietal cortex consistent with the findings in Kret et al. (2011a). To summarize, this univariate analysis including three stimulus types and four emotion categories replicates some main findings about brain areas involved, respectively, in face, body and voice expressions.

### Multivariate analysis

The goal of our multivariate approach was to reveal the areas that contribute most strongly to an accurate distinction between the modalities and the stimulus emotion. Our MVPA searchlight analysis results show that stimulus modality can be decoded from the early sensory cortices and that emotion can be decoded in STG for voice stimuli with relatively high accuracy. In STS, cingulate and angular gyrus emotion could be decoded for face and body stimuli but only with low accuracies and lenient thresholding at the group level. On the other hand, we could not clearly identify supramodal emotion regions, defined by voxel patterns where emotion could be decoded and that would show very similar voxel patterns for the same emotion in the different modalities. This indicates that the brain responds to facial, body and vocal emotion expression in a sensory specific fashion. Thus, the overall direction pointed to by our results seems to be that that being exposed to emotional stimuli (that are not task relevant and while performing a task requiring attention to the other modality than that in which the stimulus is presented) is associated with brain activity that shows both an emotion specific and a stimulus and modality specific pattern.

### ROI analysis

To follow up on the whole-brain analysis we performed a detailed and specific analysis of several ROIs. For the ROIs based on the localizer scans (early visual areas as well as FFA and EBA) stimulus type could be decoded and these results are consistent with the MVPA searchlight analysis. The A1 ROI showed above chance emotion decoding (from all stimuli or within a specific stimulus type/session). However, this region also displayed strong stimulus type decoding with no evidence of supramodal representations. Additionally, a strong effect of emotion is seen in the RDM plots in Figure 2; however, a correlational structure relating to emotion is not clearly visible within the stimulus type/session blocks. These results, together with the searchlight results, lead us to reject the hypothesis that the human brain has stimulus modality or type invariant representations of basic emotions.

### Modality specific emotion representations

Two previous MVPA studies addressed partly similar issues investigated in this study using faces, bodies and voices (Peelen et al., 2010) or bodies and voices and MVPA (Whitehead and Armony, 2019). The first study reported medial prefrontal cortex (MPFC) and posterior superior temporal cortex as the two areas hosting abstract supramodal emotion representations. These two areas are not found in our MVPA searchlight analysis. To understand the present result, it is important to remember that in the above studies participants were instructed to label the perceived emotional expressions. One motivation was that explicit judgments would increase activity in brain regions involved in social cognition and mental state attribution (Peelen et al., 2010). In contrast, the motivation of the present study was to approximate naturalistic perception conditions where people often act before and independently of tagging a label on their experience. Our design and task were intended to promote spontaneous non-focused processes of the target stimuli and did not promote amodal conceptual processing of the emotion content. It is likely that using an explicit recognition task would have activated higher level representations, e.g., orbitofrontal cortex, posterior STS, prefrontal cortex and posterior cingulate cortex that would then feed back to lower level representations and modulate these toward more abstract representations (Schirmer and Adolphs, 2017). Note that no amygdala activity was reported in that study. The second study using passive listening or viewing of still bodies and comparing fear and neutral expressions also concludes about a distributed network of cortical and subcortical regions responsive to fear in the two stimulus types they used (Whitehead and Armony, 2019). Of interest is their finding concerning the amygdalae and fear processing. While in their study this is found across stimulus type for body and voice, the classification accuracy when restricted to the amygdalae was not significantly above chance. They concluded that fear processing by the amygdalae heavily relies on contribution of a distributed network of cortical and subcortical structures.

Our findings suggest a novel perspective on the role of the different sensory systems and the different stimulus categories that convey affective signals in daily life. Paying attention to sensory specificity of affective signals may reflect better the role of emotions as seen from an evolutionary perspective and it is compatible with an ecological and context sensitive approach to brain organization (Cisek and Kalaska, 2010; Mobbs et al., 2018; de Gelder and Poyo Solanas, 2021). For comparison, a similar approach not to emotion concepts but to cognitive concepts was argued by Barsalou et al. (2003). This distributed organization of emotion representation may be more akin to what is at stake in the daily experience of affective signals and how they are flexibly processed for the benefit of ongoing action and interaction in a broader perspective of emotions as states of action readiness (Frijda, 2004).

Our results are relevant for two longstanding debates in the literature, one on the nature and existence of abstract emotion representations and basic categories and the other on processes of multisensory integration. Concerning the first one, our results have implications for the debate on the existence of basic emotions (Ekman, 2016). Interestingly, modality specificity has rarely been considered as part of the issue as the basic emotion debate largely focusses on facial expressions. The present results might be viewed as evidence in favor of the view that basic emotions traditionally understood as specific representations of a small number of emotions with an identifiable brain correlate (Ekman, 2016) simply do not exist but that these are cognitive-linguistic constructions (Russell, 2003). On the one hand, our results are consistent with critiques of basic emotions theories and meta-analysis (Lindquist et al., 2012) as we find no evidence for representations of emotions in general or specific emotions within or across modality and stimuli. Affective information processing thus appears not organized as categorically, neither by conceptual emotion category nor by modality, as was long assumed. Emotion representation, more so even than object representation, may possibly be sensory specific or idiosyncratic (Peelen and Downing, 2017) and neural representations may reflect the circumstances under which specific types of signals are most useful or relevant rather than abstract category membership. This pragmatic perspective is consistent with the notion that emotions are closely linked to action and stresses the need for more detailed ethological behavior investigations (de Gelder, 2016).

Additionally, the notion that supramodal representations of basic emotions are the pillars of emotion processing in the brain and are the basis allow of smooth translation and convergence between the different sensory modalities is not fully supported by the literature. First, since the original proposal by Ekman (1992) and the constructivist alternative argued by Russell (2003) and most recently Lewis et al. (2010) and Barrett (2017), the notion of a set of basic emotions with discrete brain correlates continues to generate controversy (Kragel and LaBar, 2016; Saarimaki et al., 2016). Second, detailed meta-analyses of crossmodal and multisensory studies, whether they are reviewing the findings about each separate modality or the results of crossmodal studies (Dricu and Fruhholz, 2016; Schirmer and Adolphs, 2017), provide a mixed picture. Furthermore, these meta-analyses also show that several methodological obstacles stand in the way of valid comparisons across studies. That is, taking into account the role of task (incidental perception, passive perception, and explicit evaluation of emotional expression) and the use of appropriate control stimuli limits the number of studies that can validly be compared. Third, findings from studies that pay attention to individual differences and to clinical aspects reveal individual differences in sensory salience and dominance in clinical populations, for example in autism and schizophrenia. For example Karle et al. (2018) report an alteration in the balance of cerebral voice and face processing systems and attenuated face-*vs*-voice bias in emotionally competent individuals. This is reflected in cortical activity differences as well as in higher voice-sensitivity in the left amygdala. Finally, even granting the existence of abstract supramodal representations—presumably in higher cognitive brain regions—it is unclear how they relate to earlier stages of affective processing where the voice, the face and the body information are processed by different sensory systems comprising distinct cortical and subcortical structures. Based on previous literature general, supramodal or “abstract” representations might have been expected in brain regions such as the orbitofrontal and anterior cingulate cortex. But as noted above, these are most often reported in studies asking participants for explicit recognition and decisions on emotion categories. The modality-specific activations that were can thus not be compared with activations for abstract categories but they are interesting by virtue of their horizontal differences between each other.

It is important to make a clear distinction between supramodal perception and multisensory integration. Our study did not focus on multisensory perception, but our findings may have implications for theories of multisensory integration by challenging a strict hierarchical model. Studies reaching beyond facial expressions have primarily been motivated how the same emotion as defined by the facial expression may be communicated by different stimulus types. Our group has initiated studies on multi-stimulus and multi-modal perception and found rapid and automatic influence of one type of expression on another [face and voice (de Gelder et al., 1999); face and body (Meeren et al., 2005); face and scene (Righart and de Gelder, 2008; Van den Stock et al., 2013); body and scene (Van den Stock et al., 2014); auditory voice and tactile perception (de Borst and de Gelder, 2017)]. These original studies and subsequent ones (Müller et al., 2012) investigated the impact of one modality on the other and targeted the area(s) where different signals converge. For example, Müller et al. (2012) report posterior STS as the site of convergence of auditory and visual input systems and by implication, as the site of multisensory integration. Different affective expression signals may have horizontal and context sensitive links rather that connections that presuppose abstract emotion representations.

### Limitations

Our motivation to include three stimulus categories led to some limitations of the current design because two separate scanning sessions were required to have the desired number of stimulus repetitions. To avoid that the comparison of representations of stimuli from two different sessions was biased by a session effect, we did not include any results that referred to differences or commonalities of stimuli from different sessions (e.g., bodies vs. faces). Another possible limitation of our study is the relatively small sample but this is compensated for by the use of very sensitive methods (MVPA and crossvalidation). Furthermore, a familiar difficulty in investigating the processing of high-order emotion perception is the relation between low-level stimulus properties (in terms of spatial and temporal statistics) and higher order emotion categories. Conversely, human detection of emotion in visual or auditory samples might be based on low-level spatio-temporal properties, and matching samples for these properties might result in unnatural appearing stimuli. To remain with the characteristics derived from the expression production we used the vocalizations as they were produced together with the face and body expressions. As they were not controlled for low-level acoustic features across emotions, our results from the decoding of emotion from the voice stimuli may partly reflect these differences. But a proper control for low level features in turn requires a better understanding of the relative (in)dependence between lower and higher-level features. Presumably bottom-up and top-down interactions determine the course of affect processing as seen for visual features of whole body expressions (Vaessen et al., 2019; Poyo Solanas et al., 2020). An analysis of low-level stimulus characteristics (see Supplementary Figure S3) did not reveal strong correlations between emotion category and features. Conversely, this analysis revealed that within category (possibly due to different actors) and between emotion category variances were similar.

### Conclusion

Our results show that the brain correlates of emotional signals from the body, the face of the voice are specific for the modality as well as for the specific stimulus. These findings underscore the importance of considering the specific contribution of each modality and each type of affective signal rather than only their higher order amodal convergence possibly related to explicit recognition task demands. We suggest that future research may investigate the differences between the emotion signals and how they are complementary as a function of the context of action and not only at abstract, amodal similarity. Another source of representational variability that would need to be addressed is whether under natural conditions, the sensory modality carrying emotion information has its own preferred functionality such that, e.g., fear would more effectively conveyed by the face, anger by the body and happiness by the voice. If so, brain representation of emotions would be characterized by specific emotion, modality and spatial context combinations and behavioral relevance (Downar et al., 2001, 2002). These are highly relevant considerations for a future neuroethologically grounded research program that should start from detailed behavioral observations of how face, body, and voice expressions function in naturalistic contexts.

## Data availability statement

The raw data supporting the conclusions of this article will be made available by the authors, without undue reservation.

## Ethics statement

The studies involving human participants were reviewed and approved by FPN-Maastricht University. The ethics committee waived the requirement of written informed consent for participation.

## Author contributions

MV, KV, and BD analysis, writing, editing. All authors contributed to the article and approved the submitted version.

## Footnotes

^1^ https://www.brainvoyager.com/

^2^ https://www.fil.ion.ucl.ac.uk/spm/software/spm12/
