# Supplementary material for: Modality-specific brain representations during automatic processing of face, voice and body expressions
Source: Front Neurosci. 2023 Oct 6;17:1132088. doi: 10.3389/fnins.2023.1132088 (PMC10587395; doi:10.3389/fnins.2023.1132088)
Supplement: Supplementary file 1 [file Data_Sheet_1.docx]

**Supplementary Figure 1**

| 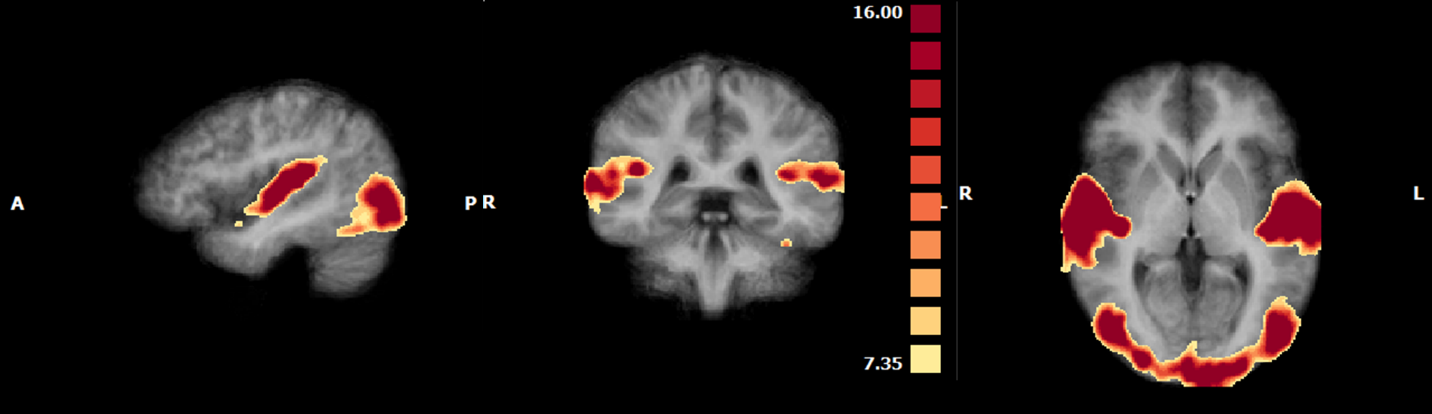 |
| --- |
| Figure S2: F-map (p<0.01 FDR corrected) for stimulus type effect. |

**Supplementary Figure 2**

| **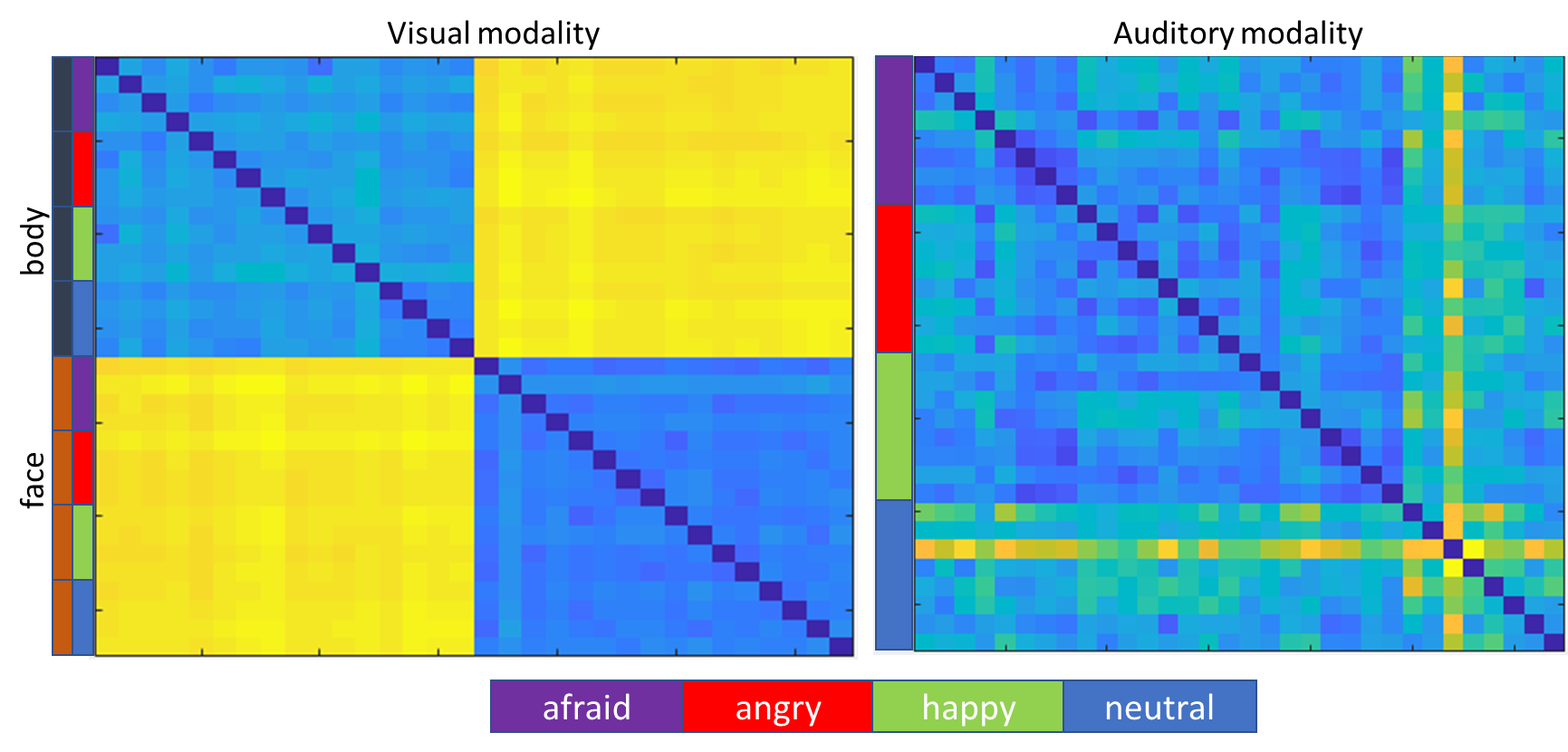** |
| --- |
| **Figure S3:** Plots showing the correlational structure of the stimuli in terms of low-level features. Left panel: the body and face videos were analysed with a Gabor pyramid model (Nishimoto et al., 2011) to yield high-dimensional descriptions of the time-frequency contents of each stimulus. These high-dimensional feature vectors were correlated for each pair of stimuli to results in the plotted representational dissimilarity matrix. Matrices are ordered by stimulus type (body or face) first and then stimulus category. Right panel: the voice stimuli were converted to spectrograms and, similarly as with the videos, theses sound features were correlated pairwise to give the RDM. Here, matrices are ordered by stimulus category. In the RDMs, blue colors indicate high similarity and yellow colors high dissimilarity. |

**Supplementary Figure 3**

| **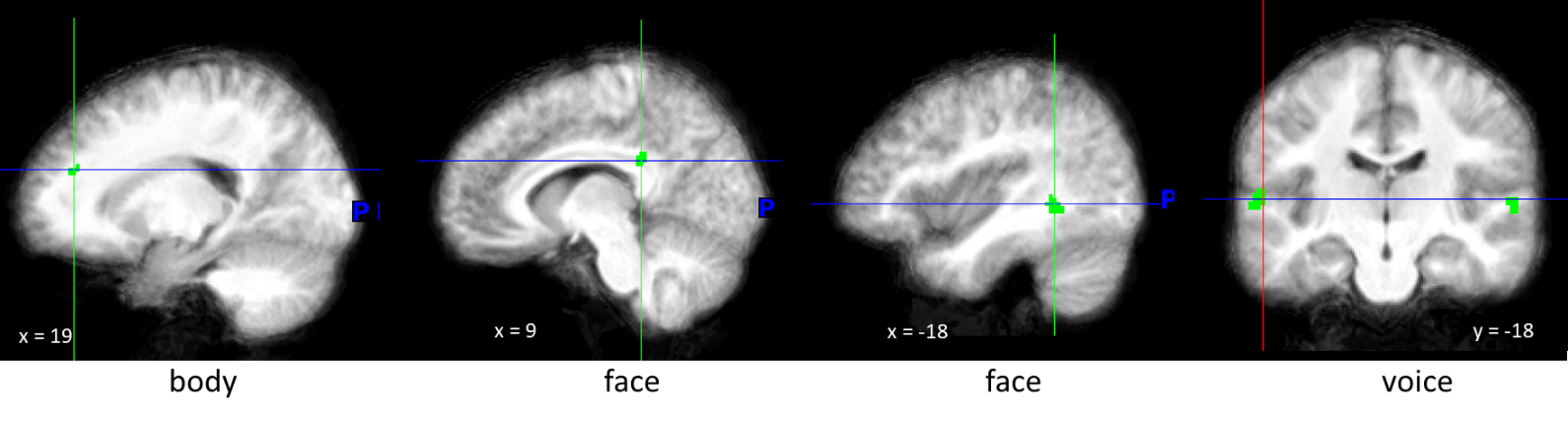** |
| --- |
| **Figure S4:** Maps showing group level inference on decoding of emotion. In green is the t-value map thresholded at t>3.9 (uncorrected) from the permutation testing for the separate modalities and stimuli types. All stimuli combined and the visual stimuli combined (face+body) did not show any supra threshold cluster and are therefore not shown. |
